# Connecting to Nature through 360° Videos during COVID-19 Confinement: A Pilot Study of a Brief Psychological Intervention

**DOI:** 10.1155/2022/4242888

**Published:** 2022-09-14

**Authors:** Jaime Navarrete, Jessica Navarro-Siurana, Rocío Herrero, Ma Dolores Vara, Marta Miragall, Rosa Baños

**Affiliations:** ^1^Polibienestar Research Institute, University of Valencia, Valencia, Spain; ^2^CIBERObn Physiopathology of Obesity and Nutrition, Instituto de Salud Carlos III, Madrid, Spain; ^3^Department of Psychology and Sociology, Universidad de Zaragoza, Teruel, Spain; ^4^Department of Personality Evaluation and Psychological Treatment Faculty of Psychology, University of Valencia, Valencia, Spain

## Abstract

Psychological interventions have been shown to be beneficial in mitigating stress related to COVID-19 confinement. According to theories of restorative environments, exposure to natural surroundings has positive effects on well-being and stress through its restorative qualities. With 360° video-based Virtual Reality (VR), people can be exposed to nature and so better manage the consequences associated with mobility restrictions during confinement. The main aim of this pilot study was to examine whether a 360° video-based VR intervention composed of five 13-minute sessions (once a day) has positive effects on affect, well-being, and stress. The sample was made up of 10 participants (4 men and 6 women; age : M = 46.5, SD = 11.7) who were confined at home (voluntarily or not) during the COVID-19 pandemic. Participants were instructed to watch a 360° video each day (of a “beach” or “lake” environment) using their smartphone and VR glasses sent to them by mail. Participants responded with several self-reports before and/or after each session (emotions and sense of presence) and before and/or after the intervention (affect, well-being, perceived stress, perceived restorativeness of nature, and the usefulness and acceptability of the intervention). Results showed a tendency to improve positive (e.g., happiness) and negative (e.g., anxiousness) emotions and experience a high sense of presence after each session. Moreover, perceived restorative qualities of the environment and their cognitive and behavioral effects were high. A significant decrease in negative affect was found after the intervention. Usefulness and acceptability were also high. This is the first study to show that an affordable and accessible technology can be used to overcome the negative consequences of confinement and counteract its harmful psychological effects.

## 1. Introduction

The COVID-19 pandemic is a very threatening event because its impact is unpredictable, long-lasting, and wide-ranging [1]. This health crisis has compelled almost every government worldwide to take extreme measures to stop the virus's spread. Quarantine has been one of the most effective and widely used measures to deal with this global situation. However, although this is not the first time this type of action has been taken (i.e., to face Severe Acute Respiratory Syndrome, SARS, [2]), the restrictions caused by COVID-19 might be some of the most extensive and intense to date, affecting almost the entire world [3].

Recent literature has shown that confinement can affect people's mental health and well-being, leading to symptoms of emotional exhaustion, irritability, insomnia, and stress, among others [1, 4–9]). Specifically, Riva and Wiederhold [10] proposed three psychological dilemmas people have to deal with in this situation: stress due to the disease, lack of access to physical places, and a sense of community crisis. This tremendous impact produces the need to ensure effective measures to mitigate stress and other related symptoms and make the situation as tolerable as possible [11, 12], while keeping in mind the limitations imposed by the quarantine restrictions. Given this scenario, technology-based interventions arise as valuable tools to promote mental health and well-being. Specifically, the use of digital technologies allows psychological interventions to be delivered in a widespread and easily accessible way while minimizing the need for mobility in these circumstances [13].

Virtual Reality (VR) can be defined as “a collection of technologies that allow people to interact efficiently with 3D computerized databases in real time using their natural senses and skills” ([14], p. 912). Of the VR systems available, mobile-based VR can be especially useful in overcoming the psychological burden of COVID-19, given its widespread accessibility and low cost [10]. In particular, exposure to nature through 360° VR videos can be a promising strategy to increase emotional well-being in people who do not have access to the outdoors due to confinement measures. Evidence shows the potential benefits of exposing individuals to nature [15, 16], especially in nature-deprived situations, such as long-term inmates in prisons and mental hospital patients, among others [17–19]. These interventions have been shown to be beneficial in increasing positive affect and decreasing negative affect and stress (e.g., [20–23]. Specifically, Attention Restoration Theory (ART; [24] has shown the potential effects of nature on stress and well-being. ART states that the perceptual experience of scenes and elements of nature (e.g., forests) facilitates recuperation from mental fatigue [25, 26] and recovery from psychophysiological stress [27]. These environments are often referred to as “restorative” because exposure to them involves the renewal of depleted cognitive resources and recovery from negative psychophysiological states [28]. Moreover, evidence has shown that natural environments have inherently restorative properties [29, 30] and capture people's fascination [31].

A recent review describes empirical studies that utilize virtual nature for restoration [32]. For instance, in a laboratory-based study, Yeo et al. [33] found that a 5-min virtual exposure to a coral reef reduced boredom and negative affect and increased positive affect and connectedness with nature. Similar results were obtained by Chung et al. [34]; who found that exposure to a 5-min 360° nature VR helped individuals suffering from mental fatigue to better manage their attentional resources. Another example of the use of the combination of VR and nature exposure to help overcome the psychological burden of COVID-19 is the weekly self-help VR protocol by Riva et al. [35]. The virtual world simulates a natural environment in order to promote relaxation and self-reflection, and the VR video is combined with daily exercises to be experienced with another person. Moreover, Browning et al. [36] compared the effectiveness of exposure to natural surroundings, exposure to a 360-degree VR nature video, and an indoor control condition (i.e., to sit in front of a blank white wall). Physiological arousal (i.e., skin conductance), restorativeness, and positive and negative affect were analyzed before and after the 6 minutes exposure. The only condition that increased positive affect was the outdoor condition, but both outdoor and VR conditions were superior to sitting indoors with no exposure to nature in terms of increasing skin conductance and restoration. Hence, exposure to nature on a mobile VR headset produced similar well-being benefits to exposure to nature outdoors.

In sum, the empirical evidence on nature's restorative effects and the potential of immersive technologies provides the basis for designing strategies that promote well-being by savoring natural scenes on mobile-based VR. However, to our knowledge, there is scant evidence showing the effect of VR-based interventions that foster exposure to nature in a confined population.

The primary aim of the present study is to examine whether brief, structured, 360° VR video training can produce restorative effects through engagement with nature. Specifically, we analyze the impact of this VR intervention on positive and negative affect, well-being, perceived stress, emotions, and perceived restorativeness in confined people. We hypothesize that this brief 360° video-based VR intervention will show a high level of the environment's perceived restorativeness, usefulness, and acceptability (hypothesis 1). Moreover, we expect that participants will experience a high level of positive emotions (happiness and calmness) and a low level of negative emotions (anxiousness, sadness, and irritation) after each session (hypothesis 2). Additionally, we hypothesize that participants will experience a high sense of presence in the virtual environments (hypothesis 3). Finally, we expect that this VR-based intervention will increase positive psychological functioning (positive affect and well-being) and reduce negative psychological functioning (negative affect and perceived stress) (hypothesis 4).

## 2. Material and Methods

### 2.1. Participants

The sample was made up of 10 participants (4 men and 6 women) between 28 and 67 years old (M = 46.5, SD = 11.7). None of them suffered from COVID-19 or had a close relative who had the illness. Sociodemographic characteristics are detailed in Table 1.

The study was advertised on social networks (e.g., WhatsApp and Instagram). Interested people contacted the researchers by email and received information about the study. After providing their sociodemographic data, those who met the inclusion and exclusion criteria were invited to participate. The inclusion criteria consisted of: (1) being confined at home (voluntarily or not) during the COVID-19 pandemic (June–August 2020), only leaving home for essential activities (such as grocery shopping and medical visits); (2) being older than 18 years old; (3) being a native Spanish speaker; (4) having a smartphone with a gyroscope and Internet connection; and (5) having an Android or iOS smartphone with the YouTube app installed and headsets with VR headset glasses. Non-voluntarily confined participants were those who lived in Spanish cities that were under self-imposed quarantine. The exclusion criteria were: having a mental disorder diagnosis at that time, hearing or visual disabilities, epilepsy, or brain damage.

Participants filled out the informed consent and a non-disclosure agreement before participating in the study, in accordance with the Declaration of Helsinki. The Ethics Committee at the University of Valencia (Spain) approved the study (register number: 1595575685780).

### 2.2. Measures

#### 2.2.1. Sociodemographic Data

Personal data include age, sex, marital status, work status, and personal situation during the quarantine.

#### 2.2.2. Perceived Restorative Qualities of the Environment

The short version in Spanish of the Perceived Restorativeness Scale (PRS, [37]) assesses an individual's perception of five restorative factors assumed to be present to a greater or lesser extent in the environment: (1) “being-away” from demands on directed attention; (2) “fascination,” which is a type of effortless attention and without limitations; (3) the “coherence” perceived in an environment (i.e., perceived as a whole with a larger organizational structure); (4) the “scope” perceived in an environment (i.e., perceived as possible to enter and spend time in); and (5) the “compatibility” between one's inclinations and the environmental demands. The items are rated on a 10-point Likert-type scale (0 = not at all, 10 = totally), with the total score ranging from 0 to 50. A high score on both scales indicates an increased level of perceived restorative properties of the environment.

#### 2.2.3. Environment's Restorative Influence on the Individual's Cognition and Behavior

The Restorativeness Scale (RS; [38]; Spanish adaptation by authors) is made up of four dimensions (emotional, physiological, cognitive, and behavioral) rated on a 9-point Likert-type scale (1 = not at all; 9 = extremely). Only two dimensions were assessed in the present study: (1) the *cognitive dimension*, where the participant rates the environment's influence on his/her cognition in terms of having a clear head, mental fatigue, soft fascination, and reflection, e.g., “My mental fatigue is decreasing” (5 items; scores range from 5 to 45); and (2) the *behavioral dimension*, where the participant has to describe the landscape's influence on his/her behavior in terms of being willing to seek out, explore, and stay in the environment, e.g., “I would like to stay here longer” (3 items; scores range from 3 to 27). A high score on both scales indicates a greater influence of the environment on the individual's cognition and behavior.

#### 2.2.4. Usefulness and Acceptability

This is an *ad-hoc* questionnaire composed of six items that assess the participant's opinion as to the usefulness (*“The training has been helpful to me*,*” “Other people in confinement might find the training useful”*) and acceptability (*“The intervention instructions are easily understood*;*” “I would like to continue exercising to increase my well-being*;*” “Connecting the mobile phone to the VR headset was difficult*;*” “When I wanted to explore the environment I succeeded”*) of the VR intervention on a 5-point Likert-type scale (1 = strongly disagree and 5 = strongly agree).

#### 2.2.5. Positive and Negative Emotions

A visual analogue scale with five items was used to measure the following emotions (VAS-Emotions): happy, sad, calm, anxious, and irritated. Participants rated to what extent they felt each emotion at that moment on a 5-point Likert-type scale (1 = not at all, 5 = extremely). The VAS is a self-reported measure that has been widely used and has shown good psychometric properties in measuring different moods or emotional states [39, 40].

#### 2.2.6. Sense of Presence

The Slater-Usoh-Steed Presence Questionnaire (SUS; [41]) was used to measure the characteristics of the VR experience (feeling of being there, realism, and involvement). This is a three-item scale with a 7-point Likert scale (1 = not at all, 7 = very much) ranging from 3 to 21, where a higher score indicates a greater sense of presence.

#### 2.2.7. Affect

The Positive and Negative Affect Scales (PANAS; [42]; Spanish adaptation, [43]) consist of two subscales with 10 items each that assess positive affect (PA) and negative affect (NA) on a 5-point Likert-type scale (1 = very little or not at all, 5 = very much). Participants rated the frequency with which they experienced both positive (e.g., “excited”) and negative (e.g., “irritable”) moods over the previous two weeks. Scores on each subscale range from 10 to 50, and higher scores represent a greater experience of positive or negative affect.

#### 2.2.8. Well-Being

The Warwick Edinburgh Mental Wellbeing Scale (WEMWBS; [44]; Spanish adaptation, [45]) contains 14 positively phrased items (e.g., “*I have been feeling optimistic about the future*”) that refer to the participant's mental well-being over the previous two weeks, rated on a 5-point Likert-type scale (1 = none of the time, 5 = all of the time), with a total score ranging from 14 to 70. Higher scores indicate a higher level of well-being.

#### 2.2.9. Stress

The Perceived Stress Scale (PSS-4; [46]; Spanish adaptation, [47]) is a 4-item questionnaire that measures the respondents' self-reported stress level by assessing their feelings and thoughts over the previous week. Answers are given on a 5-point Likert-type scale (0 = never, 4 = very often), with a total score ranging from 0 to 16. Higher scores on this scale indicate a higher level of stress.

### 2.3. Stimuli

The “Staying in TOuch with NaturE” (STONE) intervention is made up of two ultra-high-definition 360° videos (with a duration of 13′30″ each): a beach and a lake [48]. Each environment has natural environmental noises and a voice-in-off that guides the exploration and helps participants to savor the experience. The videos were retrieved from YouTube and edited by adding a narrative for each setting, which was designed by the authors to promote or increase the intensity, duration, and appreciation of the positive experience. Different details in the virtual environment were highlighted (e.g., focusing on the leaves, the waves in the sea, and the noise of the water).

### 2.4. Procedure

First, researchers mailed participants VR headset glasses that were compatible with their smartphones (Android or iOS) and detailed instructions for their use. Each participant received the VR headset glasses by mail and kept the glasses as a gift (valued at 12–20€). There was no face-to-face contact with the participants at any time. Participants received a document with a brief explanation of the benefits of exposure to nature and instructions on how to conduct the study sessions.

Second, the participants engaged in the intervention. Participants were instructed via e-mail to watch one 360° VR video session per day for five days. Thus, participants did one session per day for five consecutive days. Each session consisted of watching a 360° VR video (the “beach” or the “lake” environment), and participants could choose in each session which natural environment they wanted to experience through the video. They also completed a questionnaire on emotions (VAS) during the pre-post session (i.e., before and after watching the video), and a questionnaire on sense of presence (SUS) at post-session (i.e., after watching the video). Questionnaires on positive and negative affect (PANAS), well-being (WEMWBS), and stress (PSS-4) were completed before and after the intervention (i.e., in sessions 1 and 5), and questionnaires on restorativeness (PRS, RS), and usefulness and acceptability were completed after the intervention (i.e., in session 5).

The five sessions took place at home, at the time of the day the participant chose, trying to make it a quiet time without distractions. The web survey tool *LimeSurvey* was used to complete all the surveys in the study.

### 2.5. Research Design

This study follows a one-group pretest-posttest design, without control group, which involved a quasi-experimental design in which the outcomes of interest (i.e., PANAS, WEMWBS, PSS-4). The PRS, RS, usefulness, and acceptability were measured at post-intervention given the nature of these questionnaires and were measured twice in a single group: (1) prior to administering the STONE intervention (i.e., before session 1), (2) and after the intervention (i.e., after session 5). Hence, when the pretest and posttest scores differ significantly, then the difference could be attributed to the independent variable (i.e., the STONE intervention). Moreover, other outcomes of interest were measured twice—before each session and after each session—(VAS and SUS) in the five sessions of the intervention.

### 2.6. Data Analyses

All statistical analyses were performed using SPSS v.26. Descriptive statistics (median [*Mdn*], mean [*M*], and standard deviation [*SD*]) were calculated for all the study variables. Non-parametric statistical hypothesis tests were used to analyze the data, given the small sample size.

To test hypothesis 1 (i.e., high level of perceived restorativeness of the environment, usefulness, and acceptability), four one-sample Wilcoxon signed-rank tests were used to determine whether the participants' medians were equal to the central value within the possible range of scores of these measures (i.e., the PRS, the RS, usefulness, and acceptability). Overall, a statistically significant difference (*p* < 0.05) between the participants' median and the theoretical central value—and specifically, participants' median higher than the theoretical central value—would suggest that participants perceived the virtual environments as highly restorative, as well as the whole intervention being highly useful and acceptable.

To test hypothesis 2 (i.e., pre-post session changes in positive and negative emotions), 25 Wilcoxon signed-rank tests were used to determine if there were pre-post session changes in the participants' level of positive and negative emotions after each exposure to the natural environments (i.e., VAS-emotions: happy, calm, anxious, sad, and irritated). A statistically significant difference (*p* < 0.05) would suggest a change (i.e., an increase or decrease) in participants' level of happiness, calmness, anxiety, sadness, and irritation after the session.

To test hypothesis 3 (i.e., the high level of sense of presence in the virtual environment), five one-sample Wilcoxon signed-rank tests were used to determine whether our participants' median on the sense of presence (SUS) was equal to the theoretical central value within the possible range of scores of this measure. A statistically significant difference (*p* < 0.05) between both values—and specifically, a median higher than the theoretical central value—would suggest that the participants perceived a high level of sense of presence after each session.

Finally, to test hypothesis 4 (i.e., the increase in positive affect and well-being, and the decrease in negative affect and perceived stress after the intervention), four Wilcoxon signed-rank tests were used to determine pre-post intervention changes (i.e., PANAS, WEMWBS, and PSS-4). A statistically significant difference (*p* < 0.05) between pretest-posttest scores would suggest that participants increased or decreased their levels of affect, well-being, and stress after the intervention.

For all mentioned tests, the statistics reported are the Wilcoxon value (*Ws*) and the standardized test statistic (*z*). Moreover, the magnitude of the differences (i.e., the effect size) was estimated with *r*, which was calculated following this formula: $r\;=\;Z/\sqrt{\text{Total of observations}}$. According to Cohen [49], the effect size is small if the value of *r* varies around 0.1, medium if *r* varies around 0.3, and large if *r* varies more than 0.5.

Hypotheses 1 and 2 were tested on 10 participants; however, hypotheses 3 and 4 were performed on 9 participants because we lost data of one participant when managing the datasets of the online platform. In addition, in session 5, estimations were calculated with eight participants because one participant did not comply to the intervention schedule. However, the 10 participants adhered to the daily training instructions. There were no dropouts in the study.

## 3. Results

### 3.1. Perceived Restorativeness, Usefulness, and Acceptability of the Intervention

Descriptive statistics for perceived restorativeness, usefulness, and acceptability are shown in Table 2. The results of the one-sample Wilcoxon signed-rank tests are shown in the following paragraphs.

In the case of restorativeness, participants reported that the intervention facilitated significantly higher levels: (1) perceived restorativeness qualities of the environment (PRS questionnaire), *Ws* = 54.00, *z* = 2.70, *p*=0.007, and *r* = 0.85, given that the median was significantly higher than the central value of 25 (with a total score ranging from 0 to 50); (2) influence of the environment's restoration on the individuals' cognition (RS questionnaire), *Ws* = 55.00, *z* = 2.81, *p*=0.005, and *r* = 0.89, given that the median was significantly higher than the central value of 25 (with a total score ranging from 5 to 45); and (3) influence of the environment's restoration on individuals' behavior (RS questionnaire), *Ws* = 55.00, *z* = 2.82, *p*=0.005, and *r* = 0.89, given that the median was significantly higher than the central value of 15 (with a total score ranging from 3 to 27).

Regarding the intervention's usefulness, participants valued the training significantly higher than the central value of 3 (with a total score ranging from 1 to 5) in the following statements: (1) useful for oneself, *Ws* = 52.00, *z* = 2.57, *p*=0.010, and *r* = 0.81; (2) useful for other people in the same situation, *Ws* = 55.00, *z* = 2.89, *p*=0.004, and *r* = 0.91; and (3) willing to continue to use the intervention to increase their well-being after the study, *Ws* = 36.00, *z* = 2.59, *p*=0.010, and *r* = 0.82.

In relation to the intervention's acceptability (total score ranging from 1 to 5), participants valued the training significantly higher than the central value of 3 in the following statements: (1) They felt that they could interact and explore the virtual scenarios, *Ws* = 55.00, *z* = 2.89, *p*=0.004, and *r* = 0.91; (2) they did not experience any usability issues with the technology provided, and they had no problems connecting their mobile phones to the headsets, *Ws* = 00.00, *z* = −2.76, *p*=0.006, and *r* = −0.87; and (3) they reported that the instructions for using the VR headset glasses and for following the program were clear and helpful (*Mdn* *=* *5*), *Ws* = 55.00, *z* = 3.05, *p*=0.02, and *r* = 0.96.

### 3.2. Effect of the Individual Sessions on Changes in Positive and Negative Emotions

**Figure 1:**
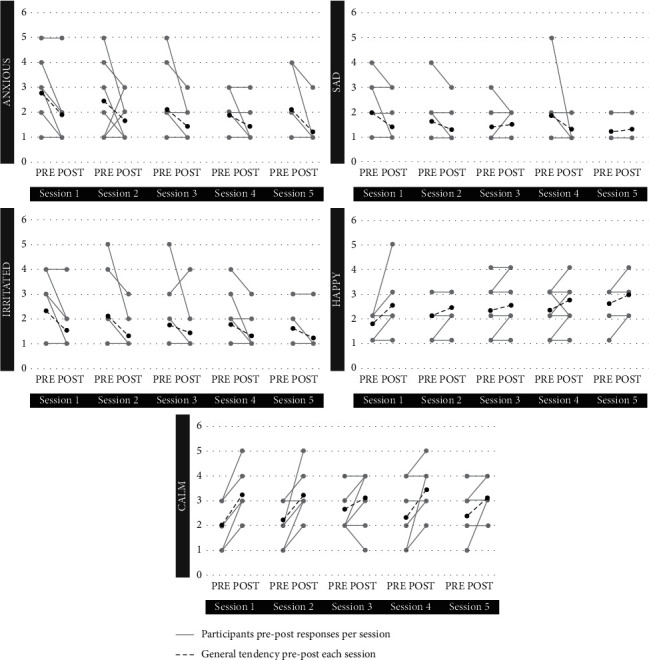
Changes in each session per emotion.

Descriptive statistics and results for the Wilcoxon signed-rank test are shown in Table 3 and Figure 1. The results revealed a statistically significant increase, with large effect sizes, in happiness in Session 1 (*r* = 0.50) and calmness in Sessions 1 (*r* = .66), 2 (*r* = 0.53), and 4 (*r* = .54). Moreover, a significant decrease was found in anxiousness in Sessions 1 (*r* = −0.54) and 5 (*r* = −0.53), and in irritation in Session 2 (*r* = −0.50). All the changes had large effect sizes. There were no statistically significant changes in the rest of the measures across the sessions (*p* > 0.05).

### 3.3. Effect of the Individual Sessions on Sense of Presence

Participants showed significantly higher rates than the central value of 12 (with a total score ranging from 3 to 21) on the sense of presence in all the sessions (SUS questionnaire), indicating that they felt present and immersed in the virtual environment during each exposure, *Ws* = 44.00, *z* = 2.55, *p*=0.011, and *r* = 0.28. The sense of presence did not decline over the sessions. Descriptive statistics are shown in Table 2.

### 3.4. Effect of the STONE Intervention on Changes in Affect, Wellbeing, and Perceived Stress

At the beginning of this study, in comparison with the normative population scores (the normative scores were: (1) WEMWBS: Castellví et al., 2014, M = 57.80 and SD = 9.40; (2) PSS-4: López-Gómez et al., 2015, M = 5.40 and SD = 2.96; and (3) PANAS: López-Gómez et al., 2015; PA, M = 32.74; SD = 8.31 and NA, M = 20.08; SD = 7.62), participants presented significantly lower scores on positive affect (PANAS questionnaire) (*Mdn* = 23.50, *M* = 24.20, and *SD* = 7.81), *Ws* = 3.00, *z* = −2.50, *p*=0.012, and *r* = .79; and well-being (WEMWBS questionnaire) (*Mdn* = 43.00, *M* = 44.40, and *SD* = 8.96), *Ws* = 00.00, *z* = −2.81, *p*=0.005, and *r* = 0.89. In addition, participants presented significantly higher scores on perceived stress (PSS-4 questionnaire) (*Mdn* = 11.00, *M* = 10.50, and *SD* = 2.46), *Ws* = 55.00, *z* = 2.81, *p*=0.005, and *r* = 0.89; however, non-significant differences in negative affect (PANAS questionnaire) (*Mdn* = 22.50, *M* = 23.70, and *SD* = 8.72), *Ws* = 38.00, *z* = 1.07, *p*=0.284, and *r* = 0.34. The one-sample Wilcoxon signed-rank test was computed with the mean of the normative data (as the median was not available). To ensure that the results were reliable, we also computed the equivalent parametric test. Results of the one-sample t-tests remained stable for positive affect, *t*(9) = −3.46, *p* = 0.007; well-being, *t*(9) = −4.73, *p* = 0.001, negative affect, *t*(9) = 1.31, *p* = 0.222, and perceived stress, *t*(9) = 60.78, *p* < 0.001).

Regarding changes in pre-post intervention scores, descriptive statistics and results for the Wilcoxon signed-rank test are shown in Table 4. Results revealed a statistically significant reduction in negative affect (PANAS questionnaire) after the intervention, with a large effect size (*r* = −0.51). There were no statistically significant changes in well-being (WEMWBS questionnaire), perceived stress (PSS-4 questionnaire), or positive affect (PANAS questionnaire) (*p* > 0.05).

**Figure 2:**
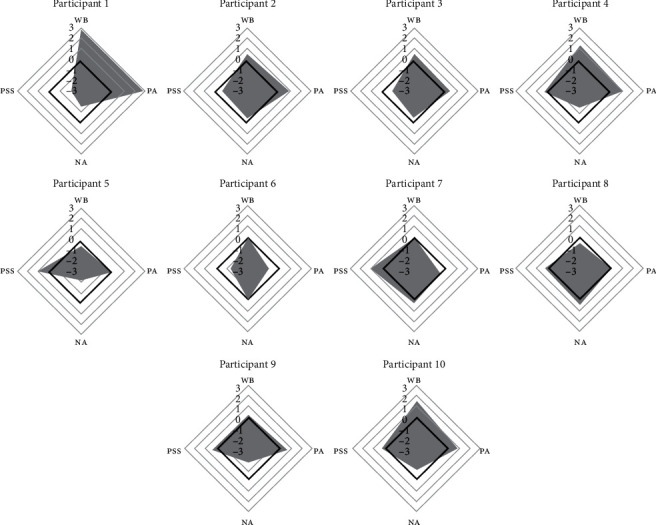
Spider chart of affect, well-being, and perceived stress difference scores (standardized scores). *Note.* PA = Positive Affect, NA = Negative Affect, WB = Well-being, and PSS-4 = Perceived Stress. The bold rhombus indicates the zero-value (the absence of change).

As shown in Figure 2, the data showed an improvement of at least one standard deviation in the positive affect scores in half of the sample (participants 1, 2, 4, 9, and 10). Three of these six participants also showed an increase in their well-being (participants 1, 4, and 10). Regarding the effect of the intervention in reducing negative affect, half of the sample showed a reduction of at least one standard deviation in their scores (participants 1, 4, 5, 9, and 10). At the same time, perceived stress decreased in four participants (participants 1, 2, 3, and 6), but it increased in two participants (participants 5 and 7).

## 4. Discussion

The present pilot study aimed to analyze the effects of a 360°-video-based VR nature intervention for people confined during the COVID-19 pandemic. Given the limited literature regarding the use of VR exposure to nature [50] and the consequences that confinement has on mental health and well-being (e.g., [1, 5, 7]), studies on the benefits of virtual exposure to nature in people who do not have access to natural environments are needed. The intervention—called STONE “Staying in TOuch with NaturE”—is based on previous evidence that has shown the restorative effect of interventions that include natural environments [15] and VR's capacity to induce and promote positive emotions and well-being [35]. In this regard, we expected to find an overall improvement in psychological functioning after administering a low-cost intervention through an easy-to-use and flexible technology in terms of technical requirements.

Specifically, our first hypothesis was supported because participants showed high levels of perceived restorativeness of the environment, usefulness, and acceptability of the VR intervention. In relation to the restorative experience, participants perceived high levels of restoration qualities in the environment (i.e., being-away, fascination, coherence, compatibility, and scope) after the intervention. Moreover, they also perceived that the environment had an effect on their cognition (e.g., mental fatigue) and behavior (e.g., desire to stay in the environment). According to ART [24] and previous studies (e.g., [51]), this finding shows that being in contact with nature is a good resource for restoring fatigued affective processes. In addition, it can be said that our settings complied with the specific elements (e.g., water features and wilderness) that natural environments need to elicit emotional states [20, 52]. Regarding the usefulness and acceptability of the intervention, participants felt that the intervention was useful—both for them and for other people—and helped them to increase their well-being. Moreover, they showed a high acceptance level because they did not experience any technical issues and they found the instructions to be clear. These aspects could explain the high adherence rate. Consequently, bearing in mind that a high percentage of the Western population has a smartphone and those participants felt the system was easy to use, our approach seems to be an inexpensive (low cost of the headsets) and acceptable way to deliver this kind of VR intervention.

Regarding our second hypothesis about the change in emotions after each session, this was partially supported. Participants significantly improved their emotional state after the first two sessions, in terms of happiness, calmness, anxiousness, and irritation, while calmness and anxiousness also improved in the last two sessions. Hence, virtual exposure was associated with effects on both positive and negative emotions, which is consistent with the effects of the 3-minute virtual exposure to coral reefs found in the laboratory study by Yeo et al. [33]. Although we did not find significant changes after each session, visual inspection of the descriptive statistics shows the participants' tendency to perceive an improvement in their emotional state after each session and a better emotional baseline before each session over time (e.g., the median for “happiness” is 2 in Sessions 1–3 and 3 in Sessions 4–5; for “anxiousness,” it is 3 in Session 1 and 2 in Sessions 2–5; for “irritation,” it is 3 in Session 1 and 1–2 in Sessions 2–5). Hence, the possibility of significant change may be limited, considering the baseline of the participants. Regarding sadness, the lack of change may be due to a “floor effect” because the sadness scores were very low before the sessions.

Moreover, our third hypothesis was supported because the STONE intervention promoted a high sense of presence in the participants. In this regard, previous studies have shown that 360° videos are effective in inducing presence (e.g., [53]). Several factors influence this variable, such as camera height or viewer position [55]. Thus, participants showed an high sense of presence even though they were not physically in the experience, which might have limited the effect of our intervention [54]. Given the small sample size, statistical analyses are limited; however, based on previous evidence (e.g., [56]), we can speculate that a sense of presence would have been essential for participants to engage in the virtual world and experience change in emotions and restoration. In the study by Yeo et al. [33], the sense of presence and connectedness to nature were the variables that mediated the effect of virtual exposure to nature on well-being. Hence, randomized controlled studies would help to disentangle the effect of our STONE intervention on the sense of presence in comparison to other technologies (e.g., computer-generated VR). In this regard, Yeo et al. [33] found that computer-generated VR was superior to 360° video in generating presence. Nevertheless, at this point, clinicians should balance the efficiency of each technology, in terms of cost-benefits, in improving the well-being of their patients.

Finally, our fourth hypothesis was that participants would show an increase in positive affect and well-being, as well as a reduction in negative affect and perceived stress after the intervention. Consistent with our hypothesis, participants experienced a significant reduction in their negative affect after the STONE intervention. This finding agrees with previous studies on the positive impact of nature experiences in reducing negative affect [57]. For instance, the study by Anderson et al. [58] has shown that exposure to 360° videos of nature reduced negative affect in comparison to a control environment. Another study revealed that VR nature exposure improved negative affect in a sample of students who experienced exam anxiety [59].

Contrary to our expectations, there were no significant changes in perceived stress. These non-significant results contrast with the systematic review by Shuda et al. [60], which concluded that exposure to nature reduced perceived and physiological stress. Several questions arise regarding the minimum doses and the frequency of being in contact with nature. In this regard, Shanahan et al. [61] pointed out that a minimum of 30 minutes per week is needed, whereas for Ulrich et al. [27], a time frame of between 5 to 7 minutes is necessary to obtain significant results. Further studies are needed to understand this unexpected result and clarify recommended frequency and minimum dose.

Similar to other studies with 360-degree videos of VR in natural environments [36, 62], we did not find a significant increase in positive affect or well-being. A recent systematic review conducted by Frost et al. [50] found that a decrease in negative affect (associated with restoration and mental fatigue recovery) is more commonly impacted by virtual immersion in nature than positive affect. In this regard, authors point out that factors that may be underlying the limited changes in positive affect during a VR nature exposure are cyber sickness, gait instability, or frustration regarding usability. Hence, future studies should control these factors to control these negative effects in increasing positive affect. Another factor may be related to the type of nature environment used in the exposure [63]. There is some evidence that high prospect (i.e., clear field of vision) and low refuge (i.e., places to hide) environments are more restorative than those with low prospect and high refuge [64]. In STONE intervention, participants chose the environment (“beach” or “lake”) in each session, but we do not consider how the participants rate the level of prospect or refuge of the chosen scenario each day. Future studies could consider the type of natural environment and compare the effectiveness of different natural elements in increasing positive affect and well-being.

### 4.1. Limitations, Strengths, and Future Directions

Several limitations should be noted. First, the sample was very small, and consequently, the effect sizes should be interpreted with caution. Second, the lack of a control group partially precludes drawing conclusions as to the effect of experimental manipulation on our outcomes. Additionally, the lack of follow-up and the length of the intervention keep us from knowing whether the effects produced are prolonged over time. Finally, another limitation is related to the confinement status and time at home of the participants. Whereas some of them were confined voluntarily, others were under mandatory confinement. Furthermore, people who were not in voluntary confinement spent a lot more time outside the house (an average of 4 hours) than people in voluntary confinement, who spent most of the day at home.

Future studies should determine the intervention's effects using a control group (e.g., playing a game with VR versus watching nature with VR) and a larger number of participants under mandatory confinement—which is associated with high psychological distress [4, 9]. This would make it possible to test whether the restorativeness of the environment (e.g., fascination) is a mediator of the effect of nature-based 360° video exposure and improvements in emotional well-being. Moreover, it would also be interesting to test the STONE intervention's effectiveness in another target population (i.e., long-term prisoners or hospital inpatients). Finally, other components could be added to this brief intervention (e.g., psychoeducational modules) to test their potential intervention effects.

Although this nature-based 360° video intervention can be improved, the STONE intervention has several strengths. On the one hand, several studies have pointed out the effects of the prolonged confinement due to the COVID-19 pandemic on mental health, but few studies have focused on potential strategies to mitigate these effects. This study shows that having contact with nature through VR may be an effective strategy to overcome the limitations on going outside during confinement periods by bringing nature back into people's lives. On the other hand, we tested the effects of repeated exposures to VR nature, whereas most studies analyze the effects of a single exposure session (e.g., a 6-minute exposure session, [36]). This is important to ensure that the effects observed after exposure to virtual nature are not only due to the novelty of VR. Finally, STONE intervention can be used on the participant's smartphone, regardless of the brand and model, with the support of affordable VR goggles. The approach used in the present study makes the intervention highly scalable and applicable in multiple contexts at a low cost and with good results and acceptable immersion levels.

## 5. Conclusion

To our knowledge, this is the first study to show the potential of using 360° video-based VR to reduce negative affect by promoting contact with nature during the COVID-19 pandemic. Moreover, the results showed a tendency to improve positive (e.g., happiness) and negative (e.g., anxiousness) emotions after each STONE session. Moreover, the 360° videos generated high levels of sense of presence, perceived restorative qualities of the environment, and perceived usefulness and acceptability by the users.

The confinement due to the COVID-19 pandemic altered daily life functioning and limited participation in activities and contexts that are usually sources of well-being, such as contact with nature. This might be particularly relevant in urban areas, where a large proportion of the population resides and where the confinement measures adopted were more rigorous. As in the proverbial phrase ‘If the mountain will not come to Muhammad, then Muhammad must go to the mountain,' we exposed a small sample of confined individuals to a lake and a beach using 360° video-based VR. The present pilot study yielded promising results with clinical implications, showing that technology-based interventions might help to overcome the limitations of lockdown and counteract their negative psychological effects.

## Figures and Tables

**Table 1:** Sociodemographic characteristics of participants (P) at the baseline.

	*P*1	*P*2	*P*3	*P*4	*P*5	*P*6	*P*7	*P*8	*P*9	*P*10
Age	57	28	47	38	34	67	41	48	57	48
Sex	Woman	Man	Woman	Woman	Woman	Woman	Woman	Man	Man	Man
Educational level	Middle	High	Low	High	High	Middle	High	High	High	High
Marital status	Married	Single	Married	Single	Married	Married	Married	Married	Married	Married
Employment	Employed	Unemployed	Unemployed	Employed	Employed	Retired	Employed	Employed	Employed	Employed
Voluntary confinement	Yes	Yes	Yes	No	No	Yes	No	Yes	Yes	No
Time at home (in hours)	—	24	23	13	19	24	18	24	24	14

*Note.* Low = Primary education; Middle = Secondary education; High = General Certificate of Education or above.

**Table 2:** Descriptive statistics of post-intervention scores (restorativeness, usefulness, and acceptability) and post-session scores for presence.

	Mdn	M	SD
Restorativeness			
Restorative experience (range: 0–50) (PRS)	41	39.20	8.70
Cognitive restoration (range: 5–45) (RS)	38	37.90	3.78
Behavioral restoration (range: 3–27) (RS)	25	24.10	2.81

Usefulness (range: 1–5)			
Useful for oneself	5	4.30	0.95
Useful for other people in the same situation	5	4.60	0.52
Willing to keep using the intervention	4	4.20	0.79

Acceptability (range: 1–5)			
Interact and explore the virtual scenarios	5	4.60	0.52
Experiencing any usability issues	1	1.50	0.71
Instructions clear and helpful	5	4.90	0.32

Presence (average sessions) (range: 3–21)	16.20	15.73	2.09
Session 1	17	15.89	2.62
Session 2	15	15.00	2.96
Session 3	17	16.00	2.45
Session 4	18	15.78	3.03
Session 5	17	16.00	2.74

*Note.* Mdn = median; M = mean; SD = standard deviation; PRS = Perceived Restorativeness Scale; RS = Restorativeness Scale.

**Table 3:** Median, mean, standard deviations, and Wilcoxon signed-rank test on pre- and post-session scores.

	Session 1 (*N* = 9)					Session 2 (*N* = 9)					Session 3 (*N* = 9)					Session 4 (*N* = 9)					Session 5 (*N* = 8)				
	Pre		Post		Z (*p*-value)	Pre		Post		Z (*p*-value)	Pre		Post		Z (*p*-value)	Pre		Post		Z (*p*-value)	Pre		Post		Z (*p*-value)
	Mdn	M (SD)	Mdn	M (SD)		Mdn	M (SD)	Mdn	M (SD)		Mdn	M (SD)	Mdn	M (SD)		Mdn	M (SD)	Mdn	M (SD)		Mdn	M (SD)	Mdn	M (SD)	
Happy	2	1.67 (0.50)	2	2.44 (1.13)	2.12 (.034)	2	2.00 (1.00)	2	2.33 (0.71)	1.73 (.083)	2	2.22 (1.09)	2	2.44 (1.13)	1.41 (.157)	3	2.22 (0.97)	3	2.67 (1.00)	1.63 (.102)	3	2.50 (0.76)	3	2.67 (1.00)	1.73 (.083)

Calm	2	2.00 (0.71)	3	3.22 (0.83)	2.81 (.005)	2	2.22 (0.83)	3	3.22 (0.97)	2.26 (.024)	3	2.70 (0.71)	3	3.11 (1.05)	1.41 (.157)	2	2.33 (1.22)	3	3.44 (0.88)	2.27 (.023)	2.5	2.38 (1.06)	3	3.11 (0.78)	1.63 (.102)

Anxious	3	2.78 (1.20)	2	1.89 (1.27)	−2.27 (.023)	2	2.44 (1.33)	1	1.67 (0.87)	−1.41 (.159)	2	2.11 (1.45)	1	1.44 (0.73)	−1.89 (.059)	2	1.89 (0.78)	1	1.44 (0.73)	−1.63 (.102)	2	2.13 (1.25)	1	1.22 (0.67)	−2.12 (.034)

Sad	2	2.00 (1.12)	1	1.44 (0.73)	−1.63 (.102)	1	1.67 (1.00)	1	1.33 (0.71)	−1.73 (.083)	1	1.44 (0.73)	2	1.56 (0.53)	0.58 (.564)	2	1.89 (1.27)	1	1.33 (0.50)	−1.34 (.180)	1	1.25 (0.46)	1	1.33 (0.50)	−1.00 (1.00)

Irritated	3	2.33 (1.32)	1	1.56 (1.01)	−1.89 (.059)	2	2.11 (1.45)	1	1.33 (0.71)	−2.12 (.034)	1	1.78 (1.39)	1	1.44 (1.01)	−0.82 (.414)	1	1.78 (1.09)	1	1.33 (0.71)	−1.63 (.102)	1.5	1.63 (0.74)	1	1.22 (0.67)	−1.73 (.083)

*Note.* Significant pre-post session changes are in bold.

**Table 4:** Median, mean, standard deviations, and Wilcoxon signed-rank test for pre- and post-intervention scores.

	Pre			Post			*Ws*	*Z*	*p*	*r*
	Mdn	M	SD	Mdn	M	SD				
Positive affect (PANAS)	23.50	24.20	7.81	29	28.30	9.13	40.00	1.28	0.201	0.28
Negative affect (PANAS)	22.50	23.70	8.72	14	17.20	7.07	5.00	−2.30	0.022	−0.51
Well-being (WEMWBS)	43	44.40	8.96	50	49.80	10.77	35.00	1.49	0.137	0.33
Perceived stress (PSS-4)	11	10.50	2.46	10	10.00	3.62	24.00	−0.36	0.719	−.08

*Note. Mdn* = median; *M* = mean; *SD* = standard deviation; *Ws* = Wilcoxon value; *z* = standardized test statistic; *p* = *p*-value; *r* = effect size estimator; PANAS = Positive and Negative Affect Scale; WEMWBS = The Warwick–Edinburgh mental well-being scale; PSS-4 = Perceived Stress Scale.

## Data Availability

The data that support the findings of this study are available in OSF at https://doi.org/10.17605/OSF.IO/SYPJF.

## Additional Points

Main contributions of the study. (i) This study examines the impact of a 360° video-based virtual reality (VR) intervention of exposure to nature (Staying in TOuch with NaturE; STONE) on well-being during COVID-19 lockdown. (ii) The STONE intervention was considered useful and easy-to-use during the lockdown. (iii) Participants felt present and immersed in the virtual environment during each exposure and attributed restorative properties to the intervention. (iv) Participants improved their emotional state after the sessions, in terms of happiness, calmness, anxiousness, and irritation. (v) The present study shows preliminary evidence of a low-cost self-applied intervention through an easy-to-use and flexible technology in terms of technical requirements.
